# Calibration of Low-Cost Sensors for PM_10_ and PM_2.5_ Based on Artificial Intelligence for Smart Cities

**DOI:** 10.3390/s26030796

**Published:** 2026-01-25

**Authors:** Ricardo Gómez, José Rodríguez, Roberto Ferro

**Affiliations:** 1 Dirección de Ingeniería Electrónica, Facultad de Ingeniería, Universidad ECCI, Bogotá 111311, Colombia; 2 Facultad de Ingeniería, Universidad Distrital Francisco José de Caldas, Bogotá 111711, Colombia; jirodriguezm@udistrital.edu.co (J.R.); rferro@udistrital.edu.co (R.F.)

**Keywords:** low-cost sensors (LCS), calibration, particulate matter (PM), machine learning, smart cities

## Abstract

**Highlights:**

**What are the main findings?**

- Improved calibration results using Fast DTW for data preprocessing.
- Comparative analysis of LCS calibration performance using statistical methods, machine learning models and deep learning.

**What are the implications of the main findings?**

- Determination of the feasibility of using calibrated LCS as a complement to traditional RMCA in the city of Bogotá.
- Application of machine learning models for LCS calibration.

**Abstract:**

Exposure to Particulate Matter (PM) is linked to respiratory and cardiovascular diseases, certain types of cancer, and accounts for approximately seven million premature deaths globally. While governments and organizations have implemented various strategies for Air Quality (AQ) such as the deployment of Air Quality Monitoring Networks (AQMN), these networks often suffer from limited spatial coverage and involve high installation and maintenance costs. Consequently, the implementation of networks based on Low-Cost Sensors (LCS) has emerged as a viable alternative. Nevertheless, LCS systems have certain drawbacks, such as lower reading precision, which can be mitigated through specific calibration models and methods. This paper presents the results and conclusions derived from simultaneous PM_10_ and PM_2.5_ monitoring comparisons between LCS nodes and a T640X reference sensor. Additionally, Relative Humidity (RH), temperature, and absorption flow measurements were collected via an Automet meteorological station. The monitoring equipment was installed at the Faculty of Environment of the Universidad Distrital in Bogotá. The LCS calibration process began with data preprocessing, which involved filtering, segmentation, and the application of FastDTW. Subsequently, calibration was performed using a variety of models, including two statistical approaches, three Machine Learning algorithms, and one Deep Learning model. The findings highlight the critical importance of applying FastDTW during preprocessing and the necessity of incorporating RH, temperature, and absorption flow factors to enhance accuracy. Furthermore, the study concludes that Random Forest and XGBoost offered the highest performance among the methods evaluated. While satellites map city-wide patterns and MAX-DOAS enables hourly source attribution, our calibrated LCS network supplies continuous, street-scale data at low CAPEX/OPEX—forming a practical backbone for sustained micro-scale monitoring in Bogotá.

## 1. Introduction

Exposure to particulate matter (PM), particularly PM_10_ and PM_2.5_, is linked with adverse human-health outcomes These manifest as an increase in respiratory conditions—such as chronic obstructive pulmonary disease (COPD), chronic cardiovascular diseases, and certain types of cancer, including lung cancer. Furthermore, exposure has been linked to a rise in premature births [1]. High concentrations of these pollutants are linked to approximately seven million premature deaths globally each year [2].

To mitigate these health risks, effective air-quality management strategies must be implemented, especially in densely populated cities. A primary strategy involves establishing Air Quality Monitoring Networks (AQMNs) comprised of fixed stations. These stations serve as the foundation for generating Air Quality Index (AQI) and providing reliable data for decision-making. However, the spatial coverage of these networks is often limited due to significant deployment and maintenance costs [3]. Specifically, a single station can cost hundreds of thousands or even millions of dollars, severely restricting the number of units that can be deployed [4].

Given the limitations of AQMNs in providing comprehensive data, the implementation of nodes based on Low-Cost Sensors (LCS) has emerged as a viable alternative. These sensors are well-suited to complement air quality monitoring in areas not covered by high-cost stations [5], thereby improving air quality characterization even where resources are limited [6].

Integrating LCS-based nodes offers distinct advantages, most notably a significant improvement in the spatial resolution of air quality data. This enhanced resolution facilitates better identification of pollution sources and trends, alongside the collection of real-time data on PM concentrations.

Nevertheless, these sensors often face accuracy challenges, including significant data outliers, weak correlations, and low data precision [7]. Additionally, inaccuracies may arise from the user’s lack of metrological knowledge, skills, and practices—a factor that can be addressed and improved through proper training in accurate data collection [8].

To place the study within its local and health context, the following is a summary of Bogotá’s atmospheric dynamics, recent PM emergencies documented by the RMCAB (Bogotá Air Quality Monitoring Network), and the relevance of the reference station in Bosa El Porvenir.

Bogotá is situated on an Andean savanna bordered by hills, with winds that favor the transport of pollutants from the north-central region to the southwest. This configuration, combined with episodes of thermal inversion and low ventilation, exacerbates PM_10_/PM_2.5_ peaks in areas such as Bosa (the area of our deployment). The RMCAB has documented two particularly severe particulate matter environmental emergencies: in 2019, and in February 2022, when smoke from regional fires combined with local sources. In both cases, alerts were declared and contingency measures were activated—strengthening controls on mobile and industrial sources, implementing restrictions and adjustments to the “peak and plate” traffic restriction/vehicle operation, and issuing communications to vulnerable populations (children, the elderly, and people with respiratory illnesses) to mitigate exposure and reduce emissions during the episode.

In this study, the placement and verification of the Local Monitoring Systems (LMS) were carried out in front of a reference station located at the Bosa El Porvenir campus of the Francisco José de Caldas District University, within the southwestern corridor where regional and urban traffic converge and where the RMCAB (Metropolitan Area of Bogotá) has reported significant PM impacts during critical episodes. We also integrated operational information from the RMCAB (meteorological conditions, stability, and PM_10_/PM_2.5_ levels) to contextualize the campaign. This combination—local reference in Bosa El Porvenir + support from the RMCAB—allows the calibration to be anchored in representative conditions and relevant epidemiological measures, aligned with the management measures that the District activates during particulate matter contingencies.

Remote-sensing approaches provide complementary perspectives to in situ sensing of particulate matter. Recent satellite methods retrieve ultrahigh-resolution PM_2.5_ at 30 m by inverting AOD from Landsat-8/Sentinel-2, enabling intra-urban hotspot mapping with cross-site accuracies around R^2^ ≈ 0.8 in urban validations; their main limitation is temporal intermittency (clear-sky, daylight overpasses), which constrains hourly and nocturnal dynamics [9]. At broader scales, global daily, gapless 1 km PM_2.5_ products fuse MAIAC-AOD with chemical-transport outputs via ensemble learning, improving continuity under clouds and supporting exposure/health assessments [9]. On the ground, MAX-DOAS hyperspectral systems resolve hectometric-scale horizontal structure on hourly cycles and are effective for source attribution and profiling of trace gases/aerosols, albeit with a limited spatial footprint around the deployment site [10]. Together, these modalities deliver wide-area coverage (satellite), daily continuity (gap-filled ensembles), and fine-scale source diagnostics (MAX-DOAS).

This study addresses the remaining gap of continuous, hyper-local monitoring in complex urban canopies. Our calibrated low-cost sensor (LCS) network provides minute-level continuity and street-scale spatial resolution at low CAPEX/OPEX, with multivariate, non-linear calibration (e.g., RF/XGBoost) that corrects hygroscopic and regime-dependent biases against the reference instrument. In practical terms, satellite products are recommended for city-wide exposure mapping and policy evaluation (30 m–1 km; cost dominated by processing), MAX-DOAS for hourly source tracking near industrial/traffic corridors (instrument + local operation), and LCS networks for operational neighborhood-level monitoring, alerts, and data fusion with satellite/MAX-DOAS when broader coverage or attribution is required. This positioning clarifies the accuracy–cost–use-case trade-offs and why the sensors developed here are the most suitable option for sustained micro-scale monitoring in Bogotá while remaining interoperable with remote-sensing layers.

This paper details the implementation of a measurement node model for Particulate Matter (PM_10_ and PM_2.5_) concentrations based on LCS. The system utilizes an ESP32 Devkit alongside Relative Humidity (RH) and temperature sensors, with measurements taken simultaneously alongside a T640X particle monitor. In total, four (4) such nodes were assembled in the city of Bogotá.

Subsequently, the paper presents the analysis of the collected data, evaluating the accuracy and effectiveness of the proposed system. This evaluation employs statistical metrics such as Mean Bias Error (MBE), Root Mean Square Error (RMSE), and the Coefficient of Determination (R^2^). The study then proceeds to present the results derived from applying machine learning techniques to the data from the simultaneous measurements, with the specific objective of calibrating the LCS.

Finally, a comparative evaluation is conducted to assess the differences between the obtained results. The findings from this evaluation will help determine the viability of using calibrated LCS as a complement to traditional AQMNs in urban settings. Furthermore, they will establish the system’s efficacy in diagnosing air quality and identifying critical areas requiring attention, thereby contributing to improved public health management in cities.

## 2. Materials and Methods

### 2.1. Monitoring Implementation and Cloud Storage

To carry out monitoring campaigns, sensor nodes were designed, implemented, and deployed. Each node is composed of a Sensirion (Sensirion AG, Stäfa, Suiza) SPS30 PM_10_ and PM_2.5_ sensor, a Sensirion SHTC3 temperature and RH sensor, an ESP32 Devkit V1 (Espressif Systems Co., Ltd., Shanghai, China) microcontroller unit (MCU), an RFM95W long-range (LoRa) HOPERF (Shenzhen Hope Microelectronics Co., Ltd., Shenzhen, China) transceiver module, and a TX915-JKD-20 LoRa (Chengdu Ebyte Electronic Technology Co., Ltd., Chengdu, China) antenna.

The sensors capture minute-by-minute readings of pollutant and meteorological parameters. These readings are retrieved via the I^2^C interface by the MCU, which appends a timestamp and node ID, packets the information, and delivers it to the LoRa module for transmission to the master node via the LoRa antenna.

Within the LoRa point-to-point LoRa link established between each sensor node and the master node, specific management functions are handled such as SF/BW/CR configuration, while collision avoidance is managed through timeslots. The resulting network follows a star topology, where the master node acts as the central hub receiving data from all peripheral sensor nodes.

**Figure 1:**
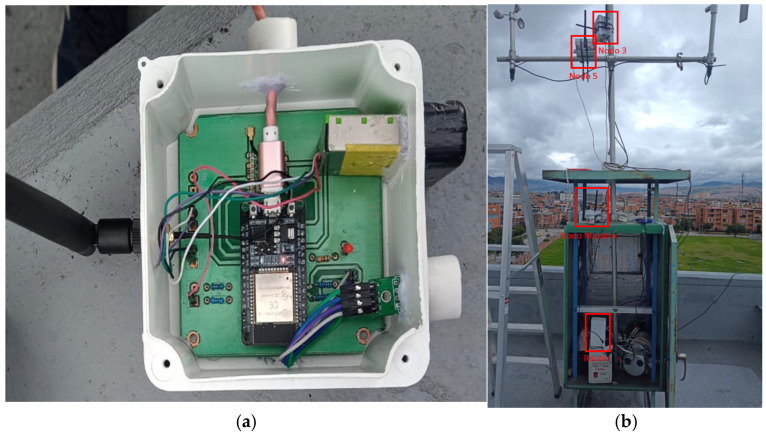
(**a**) Monitoring sensor node. (**b**) 4G router, master node and sensor nodes deployed in-site.

The master node itself is sensorless, as its primary role is to bridge within the LoRa network. To this end, it performs three key functions: first, it receives incoming data, validates data through CRC checks, recordings RSSI/SNR values, and buffers data in the event of backhaul failure; second, it establishes a connection with the Mercusys MB110-4G (MERCUSYS Technologies Co., Ltd., Shenzhen, China) Modem via Wi-Fi; and third, it maps each record to the corresponding data fields. Figure 1 shows the installed and operational setup, including two sensor nodes, the master node, and the MB110-4G modem.

The MB110-4G serves two primary functions. First, it acts as an access point and local router; in this capacity, it assigns a private IP address via DHCP, maintains a stable wireless session, and resolves the domain names (DNS) to obtain the service IP whenever the master node’s ESP32 requests to transmit data to ThingSpeak. Additionally resolves the ThingSpeak FQDN via DNS when the master node’s ESP32 initiates data uploads. It then applies NAT/PAT, translating the ESP32’s private source IP and port (e.g., 192.168.x.x:tcp/80) to the carrier-assigned public address, enabling outbound connectivity (inbound access remains blocked under 4G CGNAT unless specific tunneling/forwarding is configured).

The second function involves routing traffic to the Internet via the operator’s APN using its built-in 4G modem. To achieve this, it manages address translation (NAT), session states, and data retransmission over the mobile network. This architecture allows the ESP32 to issue IP requests without any direct interaction with the cellular layer, as the MB110-4G handles the 4G backhaul to the cloud automatically, without requiring any cellular-stack handling by the ESP32.

**Figure 2:**
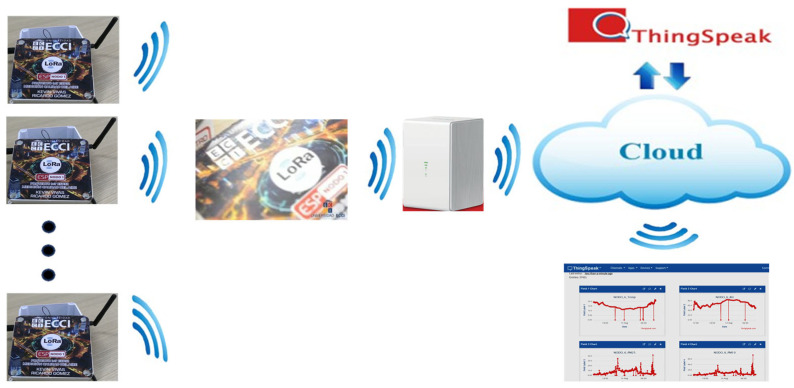
General topology of the implemented LCS monitoring network.

Figure 2 illustrates the topology of the implemented LCS network.

Finally, ThingSpeak serves as the cloud-based IoT platform where private channels ingest the measurements transmitted by the master node via a REST API, authenticated using API keys. Once the data is timestamped and stored, it can be visualized on private dashboards that support near real-time intervals.

**Figure 3:**
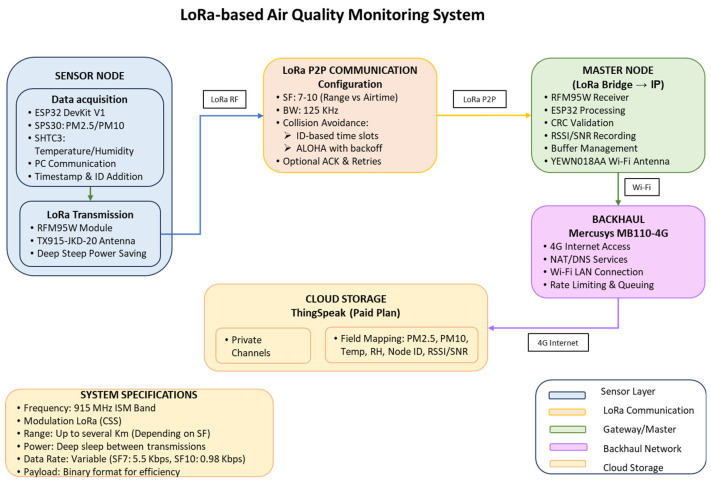
Information flow diagram of the system.

Figure 3 illustrates the flow diagram applied to the data, tracing the workflow from the deployed LCS network to its final storage in the ThingSpeak cloud.

The described LCS network conducted simultaneous monitoring campaigns alongside the Monitoring Station of the Air Quality Laboratory at Universidad Distrital, located on the rooftop of the Bosa El Porvenir campus, over a period of 2.5 months (March–May 2024). For these campaigns, the LCS network was installed at a horizontal distance of 3.5 m from and at the same height as the PM_10_ and PM_2.5_ reference sensor. The sampling interval was set to one minute, matching the period established for the reference sensor.

In addition to the Teledyne API T640X reference sensor for PM_10_ and PM_2.5_, the Universidad Distrital Air Quality Laboratory station is equipped with a Met One Automet weather station. This station monitors wind speed and direction, ambient temperature, RH, solar radiation, and precipitation.

The Teledyne API T640X is a continuous, real-time PM mass monitor that utilizes scattered light spectrometry to measure PM_2.5_, PM_10_, and coarse PM. It captures measurements at one-minute intervals and features a heater controlled to maintain 35% RH. In the U.S. EPA FRM/FEM evaluation framework, additive and multiplicative bias correspond to the linear-regression intercept (offset) and slope (gain), respectively, computed from paired candidate-method and reference-method mean measurement data [11]. Measurement artifacts associated with the Teledyne T640 under controlled chamber conditions have been reported in the literature [12].

Furthermore, the Met One Automet weather station complies with World Meteorological Organization (WMO) and EPA standards.

### 2.2. Data Preprocessing

Upon retrieval of data from both the LCS and the T640X reference sensor, data cleaning and processing were undertaken to facilitate the subsequent evaluation and correction of PM_10_ and PM_2.5_ deviations. This process was carried out through the following steps:

#### Rationale (Why These Steps)

We chose this minimal preprocessing chain to ensure temporal comparability, robustness, and numerical stability with co-located LCS models. (i) 1-min synchronization + lag correction: LCS–T640X comparison requires point-to-point correspondence; simple methods (fixed offset/correlation) do not capture variable lags, hence the subsequent use of FastDTW. (ii) IQR for outliers: It is robust to typical tailings/asymmetries in PM (better than z-score) and avoids smoothing real events (risk of median filters). (iii) Min–max [0, 1] normalization: It maintains monotonicity and improves stability in scale sensitive models (ANN) without harming trees (RF/XGB); z-score did not provide any advantage. (iv) 5000 blocks: They allow analysis of environmental regimes (e.g., RH) and drift control with bounded computational cost compared to global tuning. (v) FastDTW ≈ O(N): aligns non-constant lags with lower cost than classical DTW (O(N^2^)) and higher fidelity than a fixed shift. (vi) R^2^/RMSE/MAE metrics: cover explained variance and error magnitude with varying sensitivity to outliers; we discard MAPE/SMAPE because they are unstable at low values.

Absorption (sample) flow as covariate. The T640/T640X reports the volumetric sample flow through the optical cell; the nominal set-point is 5.0 L/min (±1%). In the T640x the total flow ≈ 16.7 L/min combines 5.0 L/min to the measurement cell plus 11.67 L/min bypass. In our data, the field Sample Flow ranged 4.95–5.05 L/min (within the set-point). Minor deviations in flow can affect inlet/conditioning efficiency and residence time especially under high RH so we normalized and included Sample Flow (L/min) as an auxiliary predictor in the multivariate calibration.

- Temporal synchronization: Measurements from the LCS and T640X were aligned using UTC timestamps at one-minute intervals. Offsets caused by optical sensor latency were corrected to ensure temporal comparability. Such co-location and temporal alignment constitute standard practice in the calibration of LCS networks.
- Outlier filtering: The Interquartile Range (IQR) method was applied to detect and eliminate spikes resulting from electronic interference and transient errors [13]. This step aligns with established QA/QC practices for PM assessment and enhances goodness-of-fit metrics [14].
- Normalization: To homogenize the scale of sensor data, PM_10_ concentrations were rescaled to the [0, 1] range using:
  (1) $$X_{norm}=\frac{X-X_{mín}}{X_{máx}-X_{mín}}$$

Normalization facilitates model training and enables comparison across devices.

- Data segmentation: The data were divided into blocks of 5000 samples, yielding 11 data sets for each of the four sensors. This approach was adopted to facilitate the analysis of environmental conditions-specifically, the impact of RH on the accuracy of PM readings-a factor identified as critical for the calibration of Low-Cost Sensors (LCS) in the reviewed literature [15].
- FastDTW application: To measure similarity between time series exhibiting potential temporal offsets, the Fast Dynamic Time Warping (FastDTW) algorithm was employed. This method serves as an efficient approximation of the classical Dynamic Time Warping (DTW) technique, which determines the optimal alignment between two series by minimizing the total distance. While DTW possesses a quadratic complexity of O(N^2^)—rendering it computationally expensive for extended sequences—FastDTW significantly reduces this complexity to approximately O(N) with minimal loss of accuracy. This enhancement is achieved through a multi-level resolution strategy, which executes DTW on progressively more detailed iterations of the time series [16]. Consequently, the implementation of FastDTW facilitates rapid and sufficiently accurate alignments suitable for the requirements of this study.
- DTW and FastDTW have also been utilized as temporal alignment tools in fields such as biomedicine, where DTW has been applied to voice signals to align patterns and generate severity biomarkers in COVID-19 patients [17]. Similarly, in finance, multidimensional DTW has been employed to identify “lead–lag” relationships between time series, underscoring its capability to align trajectories with time-varying shifts [18].
- The calculation of Statistical metrics such as R^2^ (Coefficient of Determination), RMSE (Root Mean Square Error), and MAE (mean absolute error) was performed both before and after preprocessing. This approach helped quantify the level of agreement with the T640X data. The use of these metrics follows recent recommendations for comparing co-located calibrators and is now widely adopted, as highlighted by [19,20].

These metrics are crucial for evaluating the accuracy and effectiveness of models:

○ R^2^ indicates how well the data fit a statistical model, with values close to 1 suggesting a strong fit.
○ RMSE measures the average magnitude of the errors between predicted and observed values, providing insight into the model’s accuracy.
○ MAE calculates the average absolute errors, offering a straightforward measure of prediction accuracy without emphasizing larger errors.

Their application is significant in fields that rely heavily on precise data measurement and analysis, ensuring that the calibration and data processing methods employed provide reliable and accurate results.

Equations (2)–(4) describe the calculation of the metrics used. In all of them, $y_{i}$ denotes the measurement from the reference and ${\hat{y}}_{i}$ instrument and represents the calibrated estimate from the LCS.

(2) $$R^{2}=1-\frac{\sum_{i=1}^{n}{\left(y_{i}-{\hat{y}}_{i}\right)}^{2}}{\sum_{i=1}^{n}{\left(y_{i}-\bar{y}\right)}^{2}}$$

(3) $$RMSE=\sqrt{\frac{1}{n}\sum_{i=1}^{n}{\left(y_{i}-{\hat{y}}_{i}\right)}^{2}}$$

(4) $$MAE=\frac{1}{n}\sum_{i=1}^{n}\left|y_{i}-{\hat{y}}_{i}\right|$$

### 2.3. Calibration of LCS Sensors

For the calibration of LCS sensors, a comparative analysis was conducted among six methods, from which the most suitable one was evaluated and selected: the statistical methods of Linear Regression (LR) and Generalized Regression (GR), Machine Learning techniques including Decision Trees, KNN, and XGBoost, and the Deep Learning technique ANN.

We selected two statistical approaches (LR, MLR) as interpretable and traceable baselines against the reference; three machine learning approaches (Random Forest, XGBoost, ANN) to capture nonlinearities and interactions between localized location systems (LCS) and meteorological variables; and one deep learning model (ANN) to explore temporal dependencies when memory delays/effects exist. In our co-location dataset (1 min resolution), the best R^2^, RMSE, and MAE values were obtained with Random Forest and XGBoost, which consistently outperformed LR/MLR and ANN, offering the best balance of accuracy and complexity. LR/MLR remains the reference due to its simplicity and explainability; ANN did not provide systematic improvements with the available data size; LSTM is useful when temporal memory is critical, but did not consistently improve the metrics in this study.

In terms of computational cost and deployment, LR/MLR exhibits low training and inference costs; RF/XGB require moderate training (they scale with the number/depth of trees) but maintain millisecond inference, making them suitable for periodic recalibrations and field operation; ANN/LSTM involve higher costs (and, in the case of ANN, sequence length dependence). Based on these considerations and our results, we recommend RF/XGB for the operational calibration of LCS, preserving LR/MLR as transparent baselines and reserving ANN for scenarios where explicit modeling of memory effects is required.

Model setup, data splitting, and validation. We worked with 1-min time series including PM_LCS, T, RH, and timestamps (hour/day). Imputation was applied using SimpleImputer (mean), and categories (sensor_id, time_slot) were coded using one-hot/label encoding. To maintain consistency with Section 2.2, variables were scaled using min-max (fitted in train and applied to val/test). Additionally, StandardScaler was evaluated in KNN/ANN for its suitability for distances and optimization (same no-leakage criteria: fit in train). The time-respectful split was 70/15/15 (train/val/test) in contiguous blocks, and validation was performed using blocked time-series CV (5 folds of increasing origin) selected by RMSE. For spatial robustness, Group/Spatial CV was applied (grouping by sensor_id/location), and residual autocorrelation was verified with Moran’s I. In addition, temporal blocking was used to pre-vent leakage. Hyperparameter fitting combined Randomized Search (50–100 trials) and fine grid; XGBoost and ANN used early stopping (50-s patience) while monitoring RMSE during validation. The final evaluation and reported metrics are derived from the unseen test block; in our data, Random Forest and XGBoost achieved the best R^2^/RMSE/MAE with millisecond inference latency.

Key hyperparameters (ranges and selected values).

- Random Forest (RF): n_estimators 300–600 (≈500), max_depth 10–14, min_samples_leaf 3–7, max_features = ‘sqrt’.
- early_stopping_rounds = 50.
- KNN: n_neighbors 5–15 (≈9–11), weights = ‘distance’, Euclidean metric.
- ANN (MLPRegressor): 2–3 layers (e.g., 64–32–16), activation = ‘relu’, dropout 0.1–0.3 (if applicable), Adam (lr 1 × 10^−3^), max_iter 1000, early stopping.
- (LR/MLR were used as baselines with light L2 regularization).

We trained a pooled universal model with all four LCSs simultaneously, including sensor_id as a categorical covariate (one-hot/label) along with PM_LCS, RH, T, and timestamps. Validation used Group/Spatial CV per sensor to prevent leakage between nodes. Additionally, we performed a sensitivity analysis with individual models for each sensor. The results showed comparable performance between the pooled universal approach and the per-sensor models in the unseen test block. The pooled RF/XGB universal model was preferred due to its better data utilization, robustness against inter-sensor variation, and operational transferability (same pipeline for all four nodes). Operationally, we maintain the pooled model, and if a node exhibits point drift, we apply light retuning (a short refit with its most recent co-location) without changing the overall architecture.

In addition to R^2^, RMSE, and MAE, we quantified the uncertainty of the calibrated outputs using 95% CI (from the residual variance stratified by HR and T, reporting $\hat{y}\pm 1.96{\hat{\sigma }}_{res}$ (HR,T)) and 95% PI with conformal prediction over the 15% calibration time (contrasted with quantile regression in RF/XGB for p2.5–p97.5); in the unseen test block, we observed 94–96% coverage, with wider PIs at high HR. The main sources of uncertainty include: (i) reference T640X (sample flow variation and hygrometric conditioning); (ii) LCS (noise and drift at 3–4 months; hygroscopic sensitivity at HR > 85%); (iii) temporal lag/alignment and representativeness of the sample; (iv) change in covariates between training and operation (aerosol mixture, thermal extremes); and (v) model variance (tuning/partitions). Operationally, we prioritize IP (total uncertainty) and complement it with CI (uncertainty about the conditional mean).

In all cases, data from simultaneous monitoring was used. For Machine Learning and Deep Learning techniques, the algorithms were trained using data from reference sensors while input data for obtaining metrics came from the LCS sensors. The comparison criteria were based on R^2^, RMSE, and MAE metrics, which are the most used and recommended parameters in recent LCS sensor calibration studies. For instance, ref. [21] reported improved model fit and reduced error by combining multivariate corrections with nonlinear approaches; RH is among the influential predictors commonly incorporated in recent calibration studies [22,23]. The comparative results are presented later in the Section 3.

While both Linear Regression (LR) and Generalized Regression (GR) belong to the family of linear models, there are fundamental differences in their formulation and application that justify their separate evaluation in this study: Linear Regression (LR): This is the most basic model, which assumes a direct linear relationship between predictor variables and the response variable. It operates under the assumption that errors or residuals follow a normal (Gaussian) distribution with constant variance (homoscedasticity). It is a simple and efficient model, but its performance is compromised when these assumptions are not met in the data. Generalized Regression (GR): This model, often implemented as a Generalized Linear Model (GLM), extends linear regression by allowing the response variable to follow a distribution from the exponential family (such as Binomial, Poisson, or Gamma), not just Normal. This is achieved through the use of a link function that relates the linear predictor to the meaning of the response distribution. GR is, therefore, much more flexible and robust for modeling relationships that exhibit non-normality in errors, heteroscedasticity (non-constant variance), or when the response is discrete or bounded in nature. In the context of this work, the comparison between both models is crucial. LR serves as a performance baseline. If GR offers similar or only marginally better results, it indicates that the underlying relationship between sensor measurements is essentially linear and that residuals behave approximately normally. Conversely, if GR significantly outperforms LR, it would be an indication that the particle concentration data presents characteristics (for example, a skewed distribution or a non-additive relationship) that are more effectively captured by the greater flexibility of Generalized Linear Models.

Non-linear effects of RH and temperature. The impacts of RH and T are non-linear due to known physical mechanisms: hygroscopy induces particle growth and increased optical scattering, with typical acceleration at RH ≳ 80–85% (consistent with κ-Köhler/f(RH) type formulations); in turn, T modulates the effective RH (via saturation pressure) and the partitioning of semivolatiles, generating T×HR interactions. In our data, PDP/SHAP showed marked curvature at RH and T effects mainly coupled to RH; therefore, non-linear models (RF/XGB) captured these calibration surfaces better than LR/MLR (or compact ANN), without requiring ad hoc transformations, and with inference latency compatible with operation.

Our co-location dataset covers approximately 2.5 months and therefore does not encompass a complete seasonal cycle. To mitigate this bias, we structured the analysis by weather regimes (stratification by relative humidity and temperature) and temporal validation by blocks, so that the LCS–T640X comparison was evaluated within the actually observed environmental envelope. Under these conditions, RF and XGBoost maintained the best performance in R^2^/RMSE/MAE compared to LR/MLR and ANN; however, we anticipate that extrapolation to unobserved regimes (e.g., relative humidity > 85% or temperature extremes) may degrade performance given the hygroscopic sensitivity of the optical sensors.

Operationally, we propose brief co-locations every 3–4 months (or in response to regime changes) for light recalibration, periodic verification by RH/temperature quantiles, and covariate shift detection. In multi-year deployments, we also propose expanding the evidence base with multi-season/multi-season validation and retraining guided by error thresholds. Thus, the results reported here are valid for the range of conditions covered, and the protocol explicitly outlines how to maintain performance when seasons or sites change.

## 3. Results

### 3.1. Data Preprocessing

**Figure 4:**
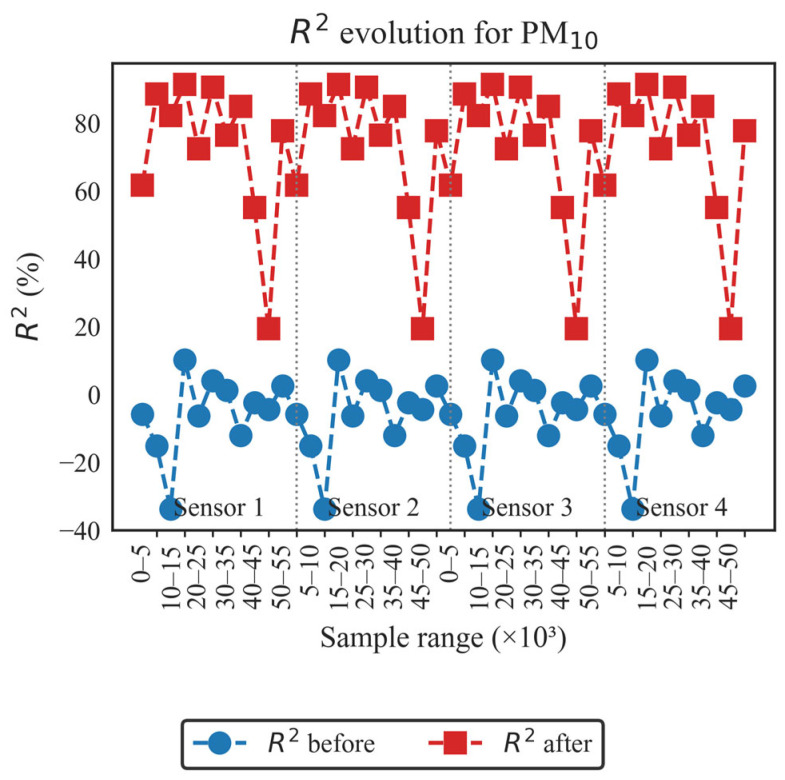
Evolution of the coefficient of determination before and after applying FastDTW for the PM_10_ concentration.

**Figure 5:**
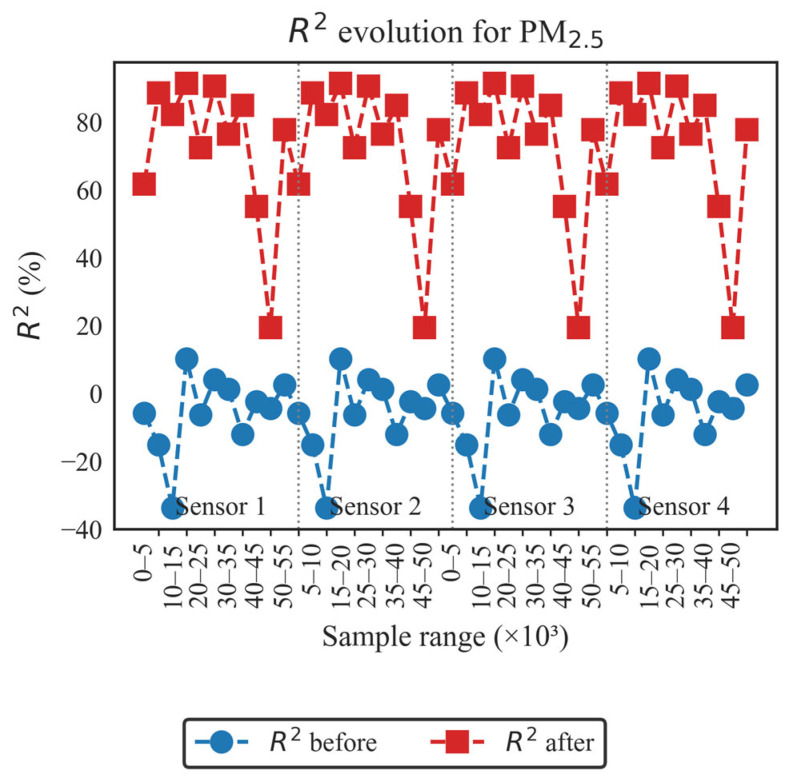
Evolution of the coefficient of determination before and after applying FastDTW for the PM_2.5_ concentration.

**Figure 6:**
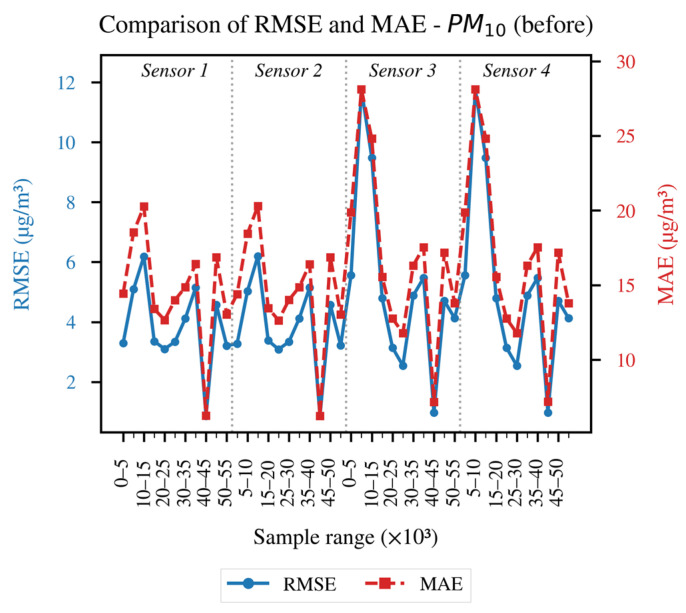
RMSE and MAE of the data before applying FastDTW for the PM_10_ concentration.

**Figure 7:**
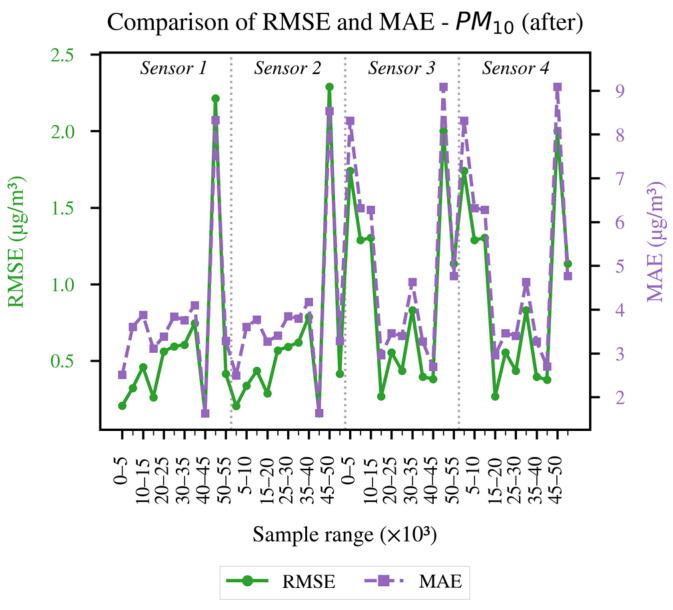
RMSE and MAE of the data after applying FastDTW for the PM_10_ concentration.

**Figure 8:**
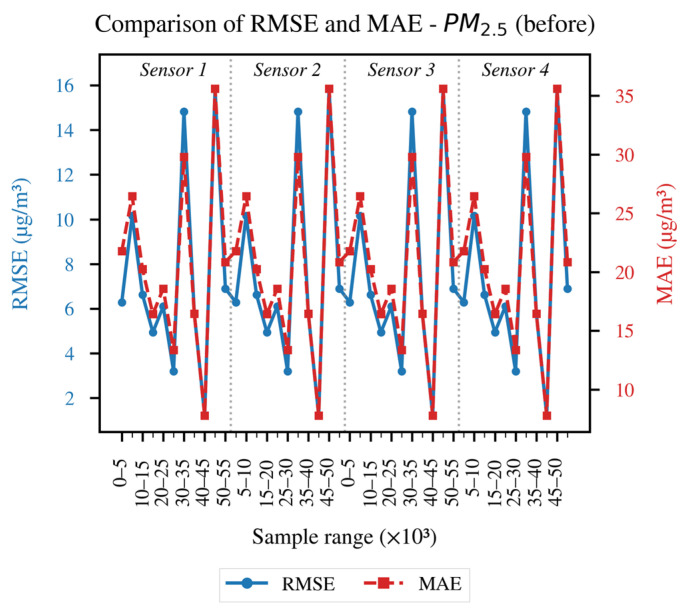
RMSE and MAE of the data before applying FastDTW for the PM_2.5_ concentration.

**Figure 9:**
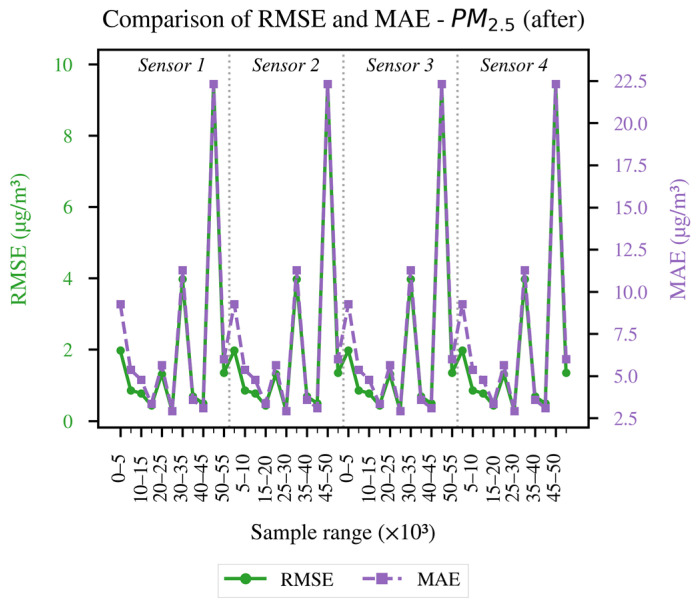
RMSE and MAE of the data after applying FastDTW for the PM_2.5_ concentration.

In order to conduct a more detailed analysis of each of the established evaluation metrics, the results obtained for each LCS in comparison with the reference sensor are presented as follows: Figure 4 and Figure 5 illustrate the evolution of the coefficient of determination (R^2^) for PM_10_ and PM_2.5_, respectively, across different data blocks; Figure 6 and Figure 7 allow us to appreciate this difference for the RMSE and MAE of PM_10_, and Figure 8 and Figure 9 do the same for PM_2.5_.

Figure 4 demonstrates that applying FastDTW consistently improves R^2^ values across all four sensors compared to the T640X. Before alignment, low and even negative R^2^ values with wide oscillations were observed, attributable to temporal desynchronization. After applying FastDTW, R^2^ values rose above 75% on average, with peaks exceeding 95%, although a drop appears in the 50k–55k block. The consistent pattern across sensors confirms that FastDTW effectively corrected temporal lags and aligned measurements to a common time scale.

In Figure 5, it can be observed that for PM_2.5_, the R^2^ behavior pattern for the four sensors is similar to that of Figure 4: a series of low “Before” values, with the particularity that they are lower than those recorded for PM_10_, and “After” series values above 70% with peaks above 85% with the same drop in the 50k–55k block. Two differences can be observed compared to PM_10_: a more sensitive drop in the 5k block, and more pronounced declines, which indicate the presence of the same meteorological factors for the four sensors.

The recorded values, for both PM_10_ and PM_2.5_, show how temporal alignment significantly reduces the decoupling, improving the calibration results, but leaving a margin that can be further improved through subsequent calibration that captures non-linear effects and the influence of meteorological factors.

As can be seen in Figure 4 and Figure 5, the performance behavior of R^2^ before and after presents a similar pattern for the four LCS sensors. As will be seen in the following figures, the performance results of RMSE and MAE before and after also present a similar pattern for the four LCS sensors.

Figure 6 presents the recorded metrics Before applying FastDTW for PM_10_. A significant variation between blocks is observed, with coinciding peaks, such as those seen in the 0k–5k and 30k–35k blocks, a situation that suggests temporal misalignments and episodes of abrupt changes in the signal. The closeness between the shapes of the curves is an indication of error distribution without heavy tails and relevant temporal biases. The maximum peaks, as mentioned earlier, indicate larger lags between the LCS and reference sensor measurements.

The recorded metrics After applying FastDTW for PM_10_ are presented in Figure 7. Both the RMSE and MAE show a decrease of an order of magnitude while maintaining the synchronization between peaks, indicators of the importance of performing temporal alignment. As with the measurements in Figure 6, there are still residual peaks in some blocks, attributable to abrupt environmental changes or differences in the response of each individual sensor. These peaks justify the performance of a subsequent calibration that includes meteorological parameters such as RH and temperature. 

Figure 8 presents the recorded results for RMSE and MAE. It can be seen that errors without alignment are more frequent, with oscillations in almost every block. This is because PM_2.5_ is more sensitive to microenvironmental conditions and temporal lags, as documented in recent research such as [15,24,25].

Figure 9 shows the RMSE and MAE values obtained after applying FastDTW. Compared with the pre-FastDTW results, both metrics decrease by roughly one order of magnitude. Additionally, the post-alignment RMSE and MAE for PM_2.5_ are generally lower than those obtained for PM_10_ after the same procedure.The magnitude of the improvement indicates that temporal decoupling is a relevant source of error; however, the presence of more noticeable residual peaks than in PM_10_ also reveals that a second calibration will be required to capture the dependencies on meteorological factors such as RH and temperature.

Overall, the results in Figure 4, Figure 5, Figure 6, Figure 7, Figure 8 and Figure 9 indicate that temporal misalignment was a significant source of error, with R^2^ values improving from low and even negative values to relatively stable ranges between 80–95% for PM_10_ and between 80–90% for PM_2.5_, with occasional block-specific drops.

Accordingly, the RMSE decreases from the range between 0.03–0.12 to the range between 0.003–0.022, and the MAE from the range between 0.10–0.28 to the range between 0.02–0.09 for PM_10_, while the RMSE mostly moves to values < 0.02 (with peaks around 0.09) and the MAE to values < 0.10 (with peaks around 0.22) for PM_2.5_.

The recorded proximity between RMSE and MAE suggests the absence of heavy tails, but the presence of residual peaks in specific ranges that are repeated in the LCS is attributable to exogenous factors such as RH and temperature, as well as rapid changes in aerosol composition or transient events.

The detailed analysis of the obtained results indicates the importance of applying FastDTW as a preprocessing step that allows establishing a common temporal structure for the set of measurements and reduces the error due to lags. However, this procedure does not completely eliminate the discrepancies, as PM_2.5_ continues to record more noticeable peaks than PM_10_, making a further processing stage necessary, in which meteorological variables such as RH and temperature are included, capturing both non-linear dependencies and scale biases. The same evaluation metrics will be applied, as they allow reflecting both the explanatory quality and the magnitude of the errors recorded in critical episodes.

The results found previously after applying FastDTW show a relatively stable behavior of the evaluation metrics, except for two blocks: the one from 40,001 to 45,000 and the one from 45,001 to 50,000, where the metrics progressively deteriorate.

**Figure 10:**
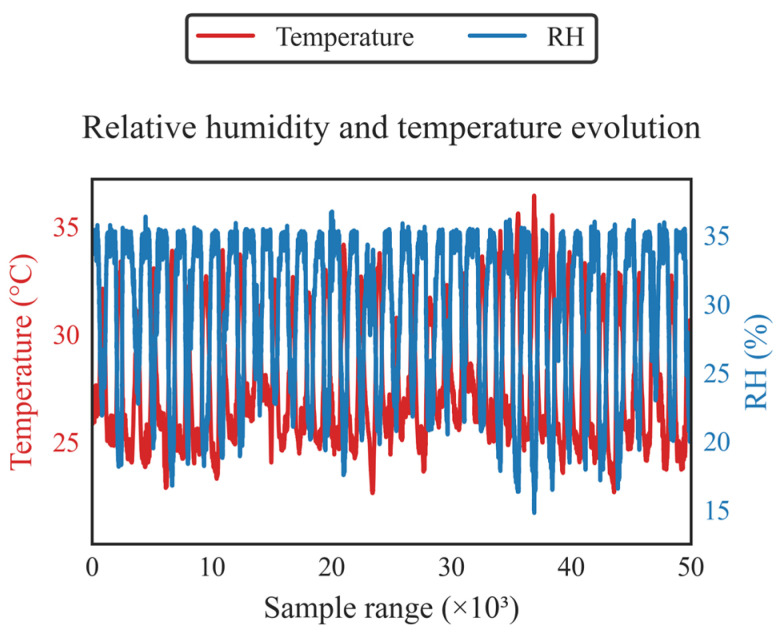
Behavior of the RH and Temperature variables, measured by the Automet weather station.

To understand what occurred, it is necessary to present the results obtained from the environmental variables RH (Relative Humidity) and Temperature, recorded by the Automet weather station, which are displayed in Figure 10.

In order to conduct a thorough analysis, it is important to note that the reference station operates with a heater controlled at 35% RH, while the LCSs (Low-Cost Sensors) lack such a device. Additionally, during the time frame corresponding to these sample blocks, both RH and Temperature exhibited unusually low values.

For the measurement block from 40,001 to 45,000, a noticeable loss of fit is indicated by R^2^ = 55.21, with upward errors reflected in RMSE = 0.49 and MAE = 0.031. This loss becomes more pronounced in the subsequent block from 45,001 to 50,000, with R^2^ dropping to 19.98, RMSE increasing to 9.37, and MAE rising to 0.221.

This behavior is evident when RH rapidly declines from the range of 35% to 38% down to 18% to 22%, while Temperature falls into the range of 2 to 4 °C. While the T640X maintains the sample at 35% RH, the LCS respond to the ambient RH, leading to a loss of hygroscopic water and a non-linear optical response that is time-dependent on the LCS. As a result, the relationship between the measurements from the LCS and the T640X breaks down, which is reflected in the deterioration of the R^2^ metric.

The degradation in the 40,001–50,000 range is explained by high/volatile relative humidity (RH), rapid transients (high dHR/dt), slight variation in sample flow around 5.0 L/min, and covariate shift (underrepresented HR–T combinations), which introduce curvature, heteroscedasticity, and variable lags that FastDTW attenuates but does not eliminate. This indicates a regime limitation (performance recovers in blocks of moderate RH). Figure 10 reinforces this diagnosis: a clear increase in RH variability and more frequent excursions are observed in the last decile (≈40–50 × 10^3^), along with inverse HR–T coupling (phase shift/anti-correlation), which implies steep flanks and a higher dHR/dt precisely where R^2^ falls and RMSE/MAE rises. As mitigation, we maintain strengthening training with high/rapidly changing HR cases and dynamic features (e.g., dew point, |dHR/dt|), explicitly including Sample Flow (L/min) and conditioning status, employing quantile-stratified HR/T validation with covariate shift detection, widening prediction bands under high HR, and performing light recalibrations every 3–4 months or after extreme episodes.

In terms of additional metrics, it is noteworthy that for the block from 45,001 to 50,000, RMSE is significantly greater than MAE, indicating the presence of high error spikes attributable to transients from dRH/dt and minor delays.

**Figure 11:**
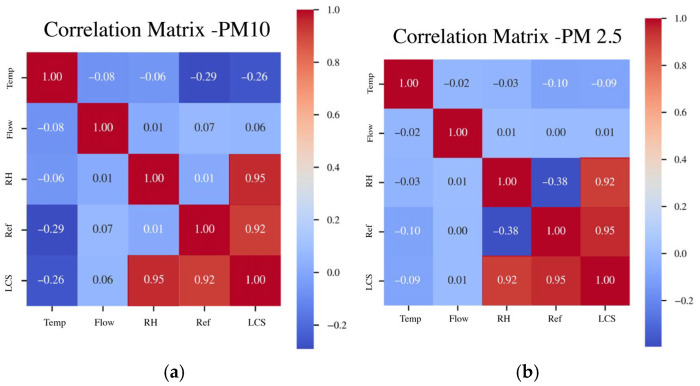
Correlation matrix between RH, Temperature, Flow and data collected by LCS and reference sensors: (**a**) for PM_10_, (**b**) for PM_2.5_.

Lastly, correlation matrices were constructed to evaluate the impact of the variables RH, Temperature, and Absortion Flow on the assessment metrics of measurements taken by the LCS and reference sensors. These matrices are presented in Figure 11.

The matrix in Figure 11a reveals a strong correlation between the measurements from the LCS and the reference sensor, with a value of 0.92, indicating that both sets of data capture a similar temporal structure. The RH is also strongly correlated with the PM_10_ sensor signals, with a correlation of 0.91 for the reference sensor and 0.95 for the LCS. This indicates that, for PM_10_, this meteorological factor induces a similar optical increase in both types of sensors. While Temperature influences the obtained measurements, it presents a moderate negative correlation (<−0.29), which is consistent with diurnal cycles where Temperature decreases and RH increases. Lastly, the Absorption Flow has a negligible impact on the results obtained.

In the case of the PM_2.5_ matrix presented in Figure 11b, a correlation of 0.95 is observed, which is even higher than that between the LCS measurements and the reference sensor for PM_10_. The correlation with RH remains similar for the LCS; however, for the reference sensor, it reaches a negative and much lower value of −0.38. This aligns with the hygroscopic bias of the LCS (over-reading) in the fine fraction compared to the result obtained with the T640X due to its drying control. In this instance, Temperature has even less influence (<0.10) compared to its effect on PM_10_ measurements. Lastly, the flow shows a negligible dependency.

These results support the findings of [26] regarding the importance of including RH as a variable in the calibration process, as well as the interactions between RH and temperature, particularly for PM_2.5_.

### 3.2. Calibration of LCS Sensors

As stated in Section 3.1, the preprocessing performed on the data significantly improves the generated metrics; however, the results obtained still show discrepancies attributable to FastDTW’s inability to capture dependencies related to meteorological factors such as relative humidity (RH) and temperature, as well as nonlinear dependencies and scaling biases.

**Figure 12:**
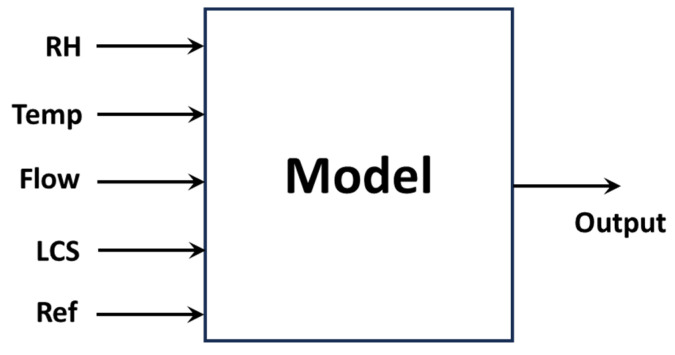
Model obtained from the analysis of the data variables, where Flow represents the absorption flow of the reference sensor, LCS are the preprocessed values from the Low-Cost Sensors, and Ref refers to the Reference Sensor.

Considering the results obtained and after analyzing them, and in accordance with the evidence found in the specialized literature, a model was developed to carry out the calibration of the LCS sensors after applying the preprocessing described in the previous section to the data. The model is presented in Figure 12 and includes meteorological input variables such as relative humidity and temperature.

To assess and compare model performance, the models and methods discussed in Section 2.3 of this article were applied to the preprocessed data, along with the other input data shown in Figure 12. The objective is to select the method that presents the best performance considering the established evaluation metrics.

**Figure 13:**
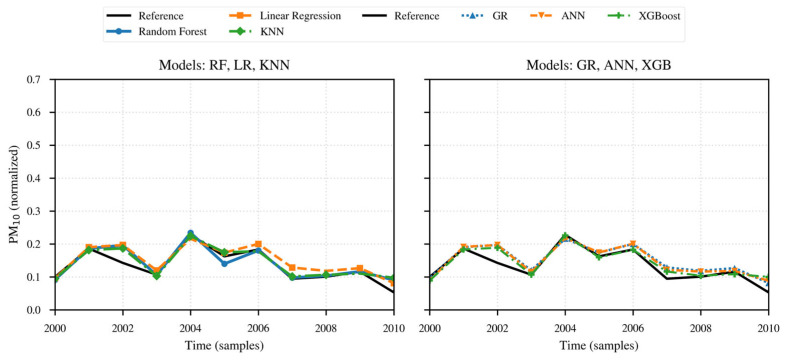
Time series of reference data and the data from the different applied models for PM_10_ concentration.

**Figure 14:**
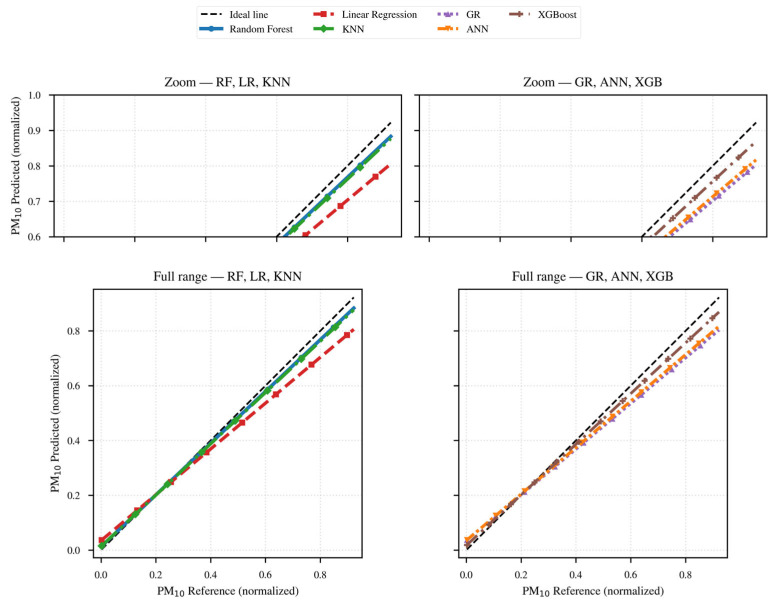
Regressions of the models applied to the PM_10_ concentration data.

For a more detailed analysis of each of the established evaluation metrics, the obtained results are presented as follows: Table 1 records the results obtained after applying the selected models and methods; Figure 13 and Figure 14 present the comparative timeline of the performance of the applied models and methods, as well as the results of the regression applied to them for PM_10_.

**Figure 15:**
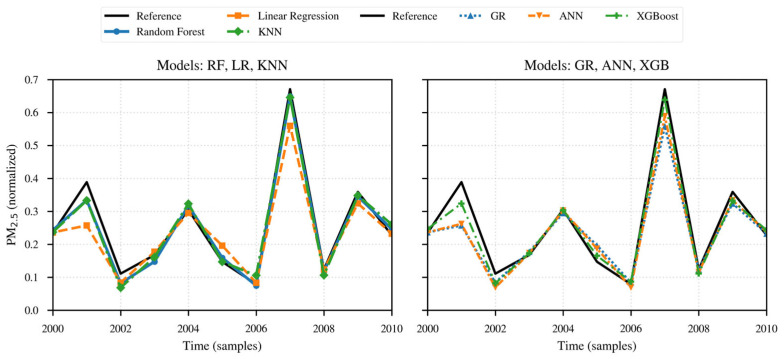
Time series of reference data and the data from the different models applied to PM_2.5_ concentrations.

**Figure 16:**
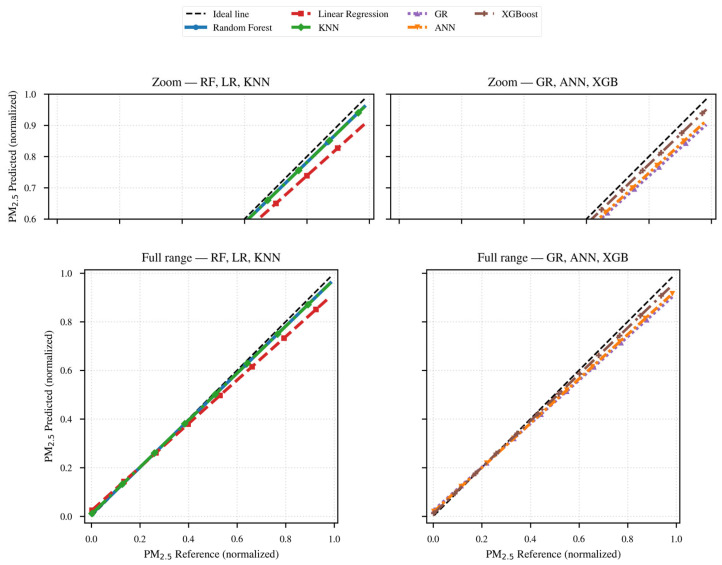
Regressions models fitted to the PM_2.5_ concentration data.

Next, The same sequence is presented for PM_2.5_, described in Table 2, and Figure 15 and Figure 16.

With the inclusion of meteorological variables, the result is that the Random Forest and XGBoost models better capture interactions and hygroscopic thresholds, with the former reflecting the lowest RMSE value (31.26) and the highest R^2^ (93.63%), while the latter achieves both the lowest MAE (0.021) and the second-best R^2^ (92.94%) and RMSE (33.95).

In the intermediate range are the KNN models, with an R^2^ of 92.14%, RMSE of 34.73, and MAE of 0.035, followed by ANN with an R^2^ of 85.51%, RMSE of 45.62, and MAE of 0.03.

Lastly, the Linear Regression and Generalized Regression methods have R^2^ values of approximately 84.05%, RMSE values around 49, and MAE values of approximately 0.048.

This indicates that a globally linear relationship does not adequately describe the bias dependent on RH and temperature, nor the nonlinearities of the LCS. The consistently high RMSE and low MAE observed across all model measurements suggest the frequent presence of peaks, which notably elevate the RMSE value.

In general, the predictions follow the temporal sequence after preprocessing, showing the most notable differences during peak events. Consistent with the obtained metrics, Random Forest and XGBoost more accurately describe sharp peaks and rapid changes, while the statistical models RL and RG tend to underestimate the crests and maintain the overall trend lines without capturing changes in sections with intermediate transitions.

KNN softens the abrupt transitions by moving away from the reference line during peaks, and ANN generally follows the reference pattern, deviating significantly during peaks and in sections with intermediate transitions.

Once again, the RF and XGBoost models cluster their estimates closer to the ideal line, with a slope slightly below 1, indicating underestimation at lower concentrations. KNN and ANN align reasonably well in the mid-range, showing greater dispersion at low values, consistent with the MAE-based results.

The RL and RG statistics show the lowest slope, indicating a systematic underestimation at higher concentration levels. This result is consistent with the findings reported in Table 1 and highlights the need to capture the nonlinear components of the interaction between the meteorological variables RH and temperature, such as hygroscopic bias.

The performance analysis of the models and methods presented for PM_10_ in the previous table and figures is consistent: the Random Forest and XGBoost methods provide the best performance because they capture the nonlinearities and interactions of the meteorological factors RH and temperature. KNN and ANN also perform acceptably but deviate from the reference pattern during abrupt transitions and peak events. The residual peaks suggest the presence of RH threshold effects and rapid episodes.

The model that achieved the best performance is Random Forest, in line with the findings reported by [27], where XGBoost was not evaluated, but KNN was, and it showed the second-best performance.

Finally, the performance analysis of the models and methods for PM_2.5_ is presented based on the evaluation metrics, following the same structure used for PM_10_, as described in Table 2 and in Figure 15 and Figure 16.

Once again, the Random Forest and XGBoost methods show the best performance, with R^2^ values of 96.52% and 96.51%, RMSE of 2.99 and 3.21, and MAE of 0.019 and 0.021, respectively.

They are followed by KNN, with an R^2^ of 95.88%, RMSE of 3.25, and MAE of 0.021, which are also acceptable results. Next are ANN and Linear Regression, with R^2^ values of 90.93% and 89.47%, RMSE of 4.84 and 2.99, and MAE of 0.028 and 0.031, respectively. Generalized Regression showed the poorest results, with an R^2^ of 89.46, RMSE of 5.28, and MAE of 0.031.

The combination of metric results obtained by Random Forest and XGBoost suggests that these models capture the interactions between RH and temperature, together with the nonlinearities in the measurements, more effectively than the other models. Finally, the relative values of the RMSE and MAE parameters indicate that Random Forest more efficiently characterizes the typical error, while XGBoost better captures the peaks.

While all the models generally reproduce the records from the reference sensor, differences arise during high-concentration episodes.

Random Forest and XGBoost provide the closest approximation in terms of periods with sharp peaks caused by rapid changes in RH and temperature. At these same peaks, both Linear Regression and Generalized Regression show crest smoothing, which corresponds to an underestimation of the peaks, mainly the lower ones.

KNN smooths very sharp transitions, with better approximation of negative peaks than the statistical regression models; for its part, ANN tends to underestimate peaks and deviate from the trend during abrupt change episodes.

Although flow does not dominate the dynamics, as seen in the correlation matrices, it helps stabilize the error when it interacts with RH and temperature during rapid events.

This diagram is consistent with those in the previous table and figure: Random Forest and XGBoost show values that are more tightly clustered around the ideal line, with a slight underestimation at the upper end in Figure 16. KNN and ANN are aligned slightly below, exhibiting heteroscedasticity at low concentrations.

The Linear and General Regression models overlap almost completely, indicating very similar values, with a systematic underestimation of data at higher values, which corresponds to a scale bias.

Clusters are observed in specific regions related to RH, which are captured more effectively by the nonlinear models.

The overall conclusion of the analysis for Table 2 and Figure 15 and Figure 16 is that the Random Forest and XGBoost models show the best performance in terms of explanatory quality and error magnitude, followed by KNN.

ANN and the statistical Linear and General models exhibit limitations such as underestimation of peaks and a lower capacity to model nonlinear interactions.

The consistency among the results considered suggests that there is still room for improvement in high-concentration episodes associated with RH thresholds and rapid changes in RH and temperature.

## 4. Conclusions

Preprocessing With FastDTW was a determining factor for carrying out the calibration of the four sensors, enabling lag correction and alignment on a common time base. This improved the statistical indicators considered for the “After” stage: R^2^ above 75% for PM_10_ and 70% for PM_2.5_, and RMSE and MAE reduced by one order of magnitude for both pollutants compared to the “Before” stage.

The analysis of the applied correlation matrices shows a strong correlation between the measurements of the LCS and the reference sensor, reaching 92% for PM_10_ and 95% for PM_2.5_. With respect to meteorological factors, a strong correlation was also found between RH and the LCS, with 95% for PM_10_ and 92% for PM_2.5_; Temperature showed a greater influence for PM_10_ (−26%) than for PM_2.5_ (9%) relative to the LCS measurements.

The analysis of the results obtained and the evidence reported in the specialized literature led to a calibration model that includes measurements from the LCS and the reference sensor, together with RH, Temperature, and the flow absorption rate of the reference sensor.

This multivariate approach enabled the assessment of RH-driven (hygroscopic) measurement bias and supported a comparative analysis of six models and methods:LR, GR, Random Forest, XGBoost, KNN, and ANN. The comparative analysis among the six models and methods was based on the evaluation metrics R^2^, RMSE, and MAE.

For both PM_10_ and PM_2.5_, the performance evaluation showed the superiority of the Random Forest and XGBoost models, as evidenced by the metrics. For PM_10_, Random Forest achieved the lowest RMSE ≈ 31 and the highest R^2^ > 93.6%, while XGBoost yielded the lowest MAE ≈ 0.021 and an R^2^ above 92.9%. For PM_2.5_, Random Forest obtained the lowest RMSE ≈ 2.99 and MAE ≈ 0.019, both with R^2^ > 96.5%.

KNN and ANN showed intermediate performance, with good temporal behavior and regressions close to the 1:1 line in the mid-range, but with dispersion at low concentrations and slight underestimation at peak values. The statistical methods of Linear Regression and Generalized Regression exhibited scale bias and high errors, suggesting a nonlinear LCS–Reference relationship across the full range.

Although both PM_10_ and PM_2.5_ achieved high R^2^ values after calibration, PM_2.5_ shows a better overall fit, as evidenced by higher R^2^ and lower MAE values, along with greater sensitivity to the meteorological factors RH and Temperature at extreme values. PM_10_, on the other hand, exhibits lower relative sensitivity with underestimation of peak concentrations.

The preprocessing and calibration phases, which included the meteorological variables RH and Temperature, identify Random Forest and XGBoost as the best-performing models, followed by KNN and ANN as alternatives with acceptable results, considering their advantages in terms of simplicity and lower computational cost. By contrast, the statistical regression models are useful for establishing a baseline for comparison but are inadequate for calibration under conditions such as those present in the monitoring performed in this study, where substantial environmental nonlinearity is observed.

The proposed methodology—preprocessing with 1-min synchronization, IQR, min-max, block segmentation, and elastic alignment, as well as RF/XGB modeling—is transferable to other urban sites if a minimal local adaptation protocol is applied: brief co-location (24–72 h) to adjust the LCS bias to the new site; covariate shift verification (quantile comparison of RH/temperature against the calibration range); fine-tuning of hyperparameters within the ranges reported for RF/XGB with block validation; and recalibration intervals of 3–4 months (or upon regime changes) to mitigate drift and seasonality. In operation, RF/XGB offers the best balance of accuracy, cost, and inference latency (ms per sample) for periodic recalibrations.

From an application standpoint, the three families of technologies are complementary: (i) satellite PM_2.5_ (30 m–1 km) supports city-wide mapping and policy assessment, with cost driven by data processing; (ii) ground-based MAX-DOAS provides hourly, hectometer-scale diagnostics suited for source tracking near corridors/emitters but with local spatial footprints; and (iii) the calibrated LCS network developed here is most suitable for operational neighborhood-level monitoring, offering minute-level continuity and low CAPEX/OPEX. In practice, these LCS nodes can serve as a high-frequency backbone and be fused with satellite/MAX-DOAS products for broader coverage and attribution.

When significantly different aerosol mixtures exist (e.g., smoke/biomass or mineral dust), extreme hygrothermal conditions (e.g., RH > 85%), or very different topographies, it is recommended to expand co-location and/or incorporate additional variables (stability indicators, source proxies) to maintain performance. We suggest publishing calibration parameters and scripts and maintaining continuous error monitoring (R^2^/RMSE/MAE by regime, drift alerts) to promote portability between stations, neighborhoods, and cities.

## Figures and Tables

**Table 1:** Statistical results after applying the models to the PM_10_ concentration.

Model	R^2^ (%)	RMSE	MAE
LR	84.0561	49.485	0.049
Random Forest	93.6363	31.263	0.031
KNN	92.1425	34.739	0.035
GR	84.0515	49.492	0.048
ANN	85.5123	47.623	0.03
XGBoost	92.9413	33.952	0.021

**Table 2:** Statistical results after applying the models to the PM_2.5_ concentration.

Model	R^2^ (%)	RMSE	MAE
LR	89.47	2.99	0.031
Random Forest	96.51	2.99	0.019
KNN	95.88	3.25	0.021
GR	89.46	5.28	0.031
ANN	90.93	4.84	0.028
XGBoost	96.52	3.21	0.021

## Data Availability

The data supporting the findings of this study are available from the corresponding author upon reasonable request.

## Abbreviations

The following abbreviations are used in this manuscript:

PM_10_	Particulate Matter 10
PM_2.5_	Particulate Matter 2.5
LCS	Low-Cost Sensors
RH	Relative Humidity
PDP	Partial Dependence Plot
SHAP	SHapley Additive exPlanations
CV	cross-validation
O(N)	Time grows linearly with N (twice the data → ~twice the time)
O(N^2^)	Twice the data → ~four times more time
MAPE	Mean Absolute Percentage Error
SMAPE	Symmetric Mean Absolute Percentage Error
LR	Linear Regression
GR	Generalized Regression
RF	Random Forest
KNN	K-Nearest Neighbors
ANN	Artificial Neural Networks
RMSE	Root Mean Square Error
MAE	Mean Absolut Error
R^2^	Coefficient of Determination
